# High-Temperature Resistant Polyborosilazanes with Tailored Structures

**DOI:** 10.3390/polym13030467

**Published:** 2021-02-01

**Authors:** Bijie Wang, Ke Chen, Tianhao Li, Xun Sun, Ming Liu, Lingwei Yang, Xiao (Matthew) Hu, Jian Xu, Liu He, Qing Huang, Linbin Jiang, Yujie Song

**Affiliations:** 1 Guangxi Key Laboratory of Petrochemical Resource Processing and Process Intensification Technology, School of Chemistry and Chemical Engineering, Guangxi University, Nanning 530004, China; wangbijie@nimte.ac.cn; 2 Engineering Laboratory of Advanced Energy Materials, Ningbo Institute of Materials Technology & Engineering, Chinese Academy of Sciences, Ningbo 315201, China; chenke@nimte.ac.cn (K.C.); litianhao2057@nimte.ac.cn (T.L.); liuming0117@hit.edu.cn (M.L.); ashxu@ntu.edu.sg (X.H.); xujian@nimte.ac.cn (J.X.); heliu@nimte.ac.cn (L.H.); huangqing@nimte.ac.cn (Q.H.); 3 Qianwan Institute of CNiTECH, Ningbo 315336, China; 4 Harbin Institute of Technology, 92 West Dazhi Street, Nan Gang District, Harbin 150001, China; sxun201@hit.edu.cn; 5 Hypervelocity Aerodynamics Institute, China Aerodynamics Research and Development Center, Mianyang 621000, China; lingwei.yang@cardc.cn; 6 School of Materials Science and Engineering, Nanyang Technological University, Block N4.1, Nanyang Avenue, Singapore 639798, Singapore

**Keywords:** polyborosilazane, boron-containing polymer, high temperature resistant

## Abstract

Boron-containing organosilicon polymers are widely used under harsh environments as preceramic polymers for advanced ceramics fabrication. However, harmful chemicals released during synthesis and the complex synthesis routes have limited their applications. To solve the problems, a two-component route was adopted to synthesize cross-linked boron-containing silicone polymer (CPBCS) via a solventless process. The boron content and CPBCSs’ polymeric structures could be readily tuned through controlling the ratio of multifunctional boron hybrid silazane monomers (BSZ12) and poly[imino(methylsilylene)]. The CPBCSs showed high thermal stability and good mechanical properties. The CPBCS with Si-H/C=C ratio of 10:1 showed 75 wt% char yields at 1000 °C in argon, and the heat release capacity (HRC) and total heat release (THR) are determined to be 37.9 J/g K and 6.2 KJ/g, demonstrating high thermal stability and flame retardancy. The reduced modulus and hardness of CPBCS are 0.30 GPa and 2.32 GPa, respectively. The novel polysilazanes can be potentially used under harsh environments, such as high temperatures or fire hazards.

## 1. Introduction

Organic–inorganic hybrid polymers have attracted much attention in recent years since they possess high-temperature resistance, anti-oxidation properties, and high mechanical strength from the inorganic segments, and processibility as well as flexibility from the organic segments [1,2,3,4,5,6]. The bottom–up approach provides enormous benefits from molecular design to tailored polymeric structures [7]. The possibility of structural design at the molecular level offers great potential in the fabrication of novel materials with desired mechanical and functional properties [8].

Organic–inorganic hybrid polymers, such as polysiloxanes, are widely used as flame-retardant materials, and molecular structure design was adopted to further enhance the flame retardancy, eliminate the use of halogen, and reduce potential environmental impacts [9,10,11,12]. Xie et al. synthesized a new phosphorus-containing ternary copolymer flame-retardant material with octa-vinyl polyhedral oligomeric silsesquioxane and poly (methyl methacrylate). The material exhibits good optical properties, impact resistance, and mechanical strength [13]. Compared with polysiloxanes, polysilazanes, which are mainly composed of Si, N, and C elements, have better mechanical properties and higher thermal stability [14,15]. The nitrogen atoms of polysilazanes could provide potential binding sites for certain chemical species [16], and the extra N-H bonds could react with Si-H or Si-vinyl bonds, allowing the further molecular design for targeted mechanical and functional properties [17]. However, polysilazanes were mostly used as the precursor of SiCN ceramics [18,19]. The one-step preparation process of polysilazanes is complicated, high cost, and it is difficult to obtain polysilazanes with high molecular weight or controlled molecular structure. The hydrolytic instability of the Si-N bonds is a bottleneck that restricts the potential applications [20,21].

Boron compounds are one class of commonly used flame retardant additives [22,23,24,25,26]. Boron can increase the crosslinking density, reduce the degree of disordered structure, and improve the heat resistance, corrosion resistance, and ablation resistance of the materials [27,28,29,30]. The incorporation of boron atoms into polysilazanes could lower their sensitivity to moisture due to reduced N-H concentration, and the synergistic effect of silicon and boron compounds provides good thermal-oxidative stability, contributing to a higher char yield, a better graphitization degree after sintering, and good flame retardancy property [8]. Yajima et al. synthesized poly(borondiphenylsiloxane) by reacting boric acid with diphenyldichlorosilane or diphenylsilane diol, and they investigated the thermal decomposition process [31]. Sundar and Keller synthesized a linear boron–silicon–diacetylene copolymer with a residual content of more than 50% at 1000 °C in air [32]. Zhou et al. synthesized linear borosilicate hybrid polymers containing C≡C units that demonstrated excellent thermal and oxidation stability [33].

However, the traditional aminolysis method used to synthesize polyborosilazanes uses environmentally hazardous chemicals (ammonia, methylamine, chlorosilazane, and pinacol borane), and the generated by-products must be specially treated. [34,35,36]. Thus, it is important to find an environmentally friendly and efficient method to synthesize polyborosilazanes.

Here, in this article, we synthesized borosilazane crosslinker via hydroboration reaction and used it to crosslink polysilazane through solventless hydrosilylation reaction without the generation of any by-products. The polymer structures and boron content could be readily tuned through adjusting the ratio of monomers used. The influence of the polymer structures on the mechanical properties and thermal properties is discussed. The design and synthesis of polyborosilazanes with tailored structures and targeted properties provide new insight into the design of new types of high-performance polymers.

## 2. Materials and Methods

### 2.1. Materials

Tetrahydrofuran (THF), borane-methyl sulfide complex (BDMS, 2.0 M solution in THF), 1,1,3,3-tetramethyl-1,3-divinyldisilazane (TMDS), and Karstedt Catalyst Solution (Pt: ≈2% Xylene solution) were purchased from Aladdin Reagent Co., Ltd. (Shanghai, China). Poly[imino(methylsilylene)] (PNSZ) was purchased from Guangzhou Winhigh Chemical & Technology Co. Ltd. (Guangzhou, China). All chemicals were used as received.

### 2.2. Preparation of BSZ12 Monomer

TMDS was dissolved in anhydrous THF under constant stirring in an ice-water bath for 20 min. BDMS was added to the TMDS solution drop by drop. The resultant solution was stirred overnight at room temperature under the protection of Argon. A transparent liquid was obtained after the solvent removal.

### 2.3. Preparation of Cross-Linked Boron-Containing Silicone Polymers (CPBCSs)

CPBCSs were prepared by mixing BSZ12, PNSZ, and Karstedt Catalyst solution (the concentration of Pt was 412 ppm) with different Si-H/C=C ratios. The samples were cured at 100 °C in air and further treated at 300 °C for 2 h under argon flow. The ratios of BSZ12 and PNSZ are listed in Table 1.

### 2.4. Characterization

^1^H-NMR and ^11^B-NMR spectra were performed inside a Bruker AVANCE III UltraShield (Bruker, Fällanden, Switzerland) spectrometer at 400 and 128 MHz, respectively. Tetramethylsilane (TMS) was used as the reference material as an internal standard in CDCl_3_ solutions at 25 °C.

Thermal analysis was performed inside a simultaneous thermal analyzer (STA; NETZSCH STA449F3, Berlin, Germany) at a heating rate of 10 °C/min under an argon and air atmosphere.

The Fourier transform infrared (FTIR) spectra were performed inside a Nicolet Avatar 360 (Thermo Fisher Scientific, Inc., Waltham, MA, USA) spectrometer using KBr disks. Spectra were recorded from 400 to 4000 cm^−1^ with 32 scans. The Ultraviolet-visible (UV-Vis) spectra were performed inside a Perkin-Elmer Lambda 950 (PerkinElmer, Waltham, MA, USA) spectrophotometer. Spectra were recorded from 200 to 800 nm.

The oxygen content and nitrogen content were determined by a LECO ON836 (LECO, Mönchengladbach, Germany), the carbon content was determined by LECO CS600, the hydrogen content was determined by an organic element analyzer (Elementar, Langenselbold, Germany), and the boron content was determined by an ICP-OES (SPECTRO ARCOS II, Kleve, Germany). The silicon content was calculated by subtracting the mass percentage of other elements.

The nanoindentation test was performed inside a NanoTest Vantage system (MML, England) equipped with a diamond Berkovich indenter. The indentation load was applied at a constant strain rate (0.05), considering the strain rate-dependent behavior of polysilazanes polymers. The peak load was set at 5 mN and maintained for 5 s. A fixed unloading time (5 s) was used for all experiments. Based on the obtained indentation force–displacement curves, the reduced modulus (*E_r_*) and hardness (*H*) were calculated based on the Oliver–Pharr method [37]. The Young’s modulus (*E*) can be finally correlated with *E_r_* by:

(1) $$\frac{1}{E_{r}}=\frac{\left(1-v^{2}\right)}{E}+\frac{1-v_{i}^{2}}{E_{i}}.$$

here, *E_i_* and *v_i_* are the Young’s modulus and Poisson’s ratio of a diamond indenter (1147 GPa and 0.07). *v* is the Poisson’s ratio of polysilazanes polymers, and an average value of 0.25 was used for all calculations.

Water contact angle analyses were performed inside a Dataphysics OCA25 using shape image analysis with 1 μL of water droplets, and the contact angle was calculated as an average of at least five samples. The surface morphologies were performed inside a Bruker Dimension icon with ScanAsyst (Bruker, Santa Barbara, CA, USA) and conducted in contact mode. The scanning sizes were 10 nm × 10 nm, and the lines are 512.

The combustibility was performed inside a pyrolysis combustion flow calorimetry (PCFC) by applying a micro-scale combustion calorimetry (MCC, Govmark MCC-2, Rochester, NY, USA) from Fire Testing Technology (FTT). First, 5 ± 1 mg of powder sample was heated under nitrogen flow up to 750 °C with a heating rate of 1 K/s. The heat release capacity (sumHRC) in J/g K was calculated by a ratio of the sum of peak HRR (pHRR—the maximum heat release rate) to the average heating rate in K/s. The total heat release (THR) in kJ/g was equal to the area below the curve of the heat release rate as a function of the pyrolysis temperature.

## 3. Results

### 3.1. Synthesis of BSZ12 Monomer and CPBCSs

**Figure 1:**
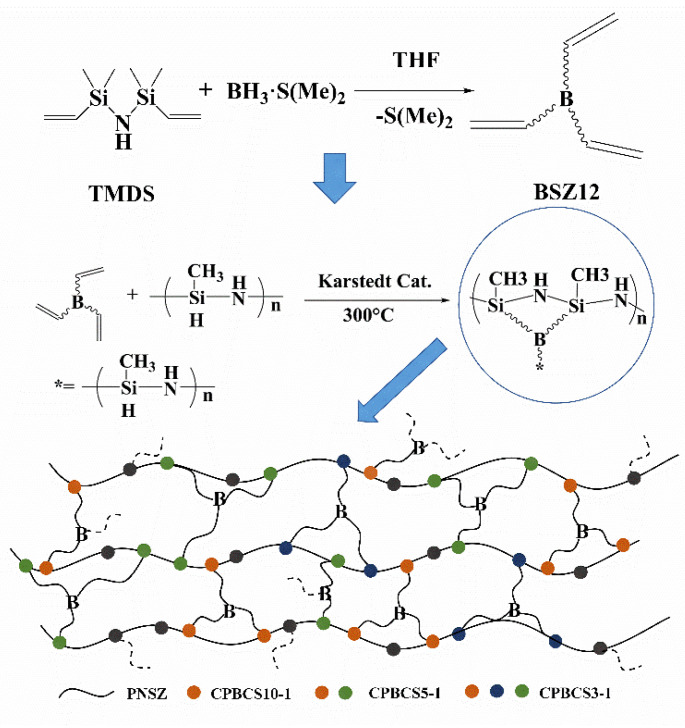
Synthetic route of boron hybrid polysilazane solid with cross-linking network (CPBCS) structure.

**Figure 2:**
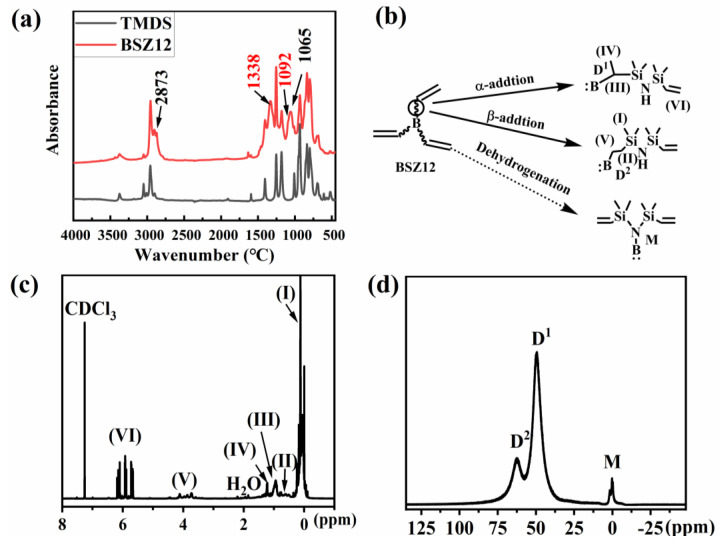
(**a**) Fourier transform infrared (FTIR) spectra of 1,1,3,3-tetramethyl-1,3-divinyldisilazane (TMDS) and BSZ12 monomers; (**b**) Structural composition of BSZ12 monomers; (**c**) ^1^H NMR spectrum, and (**d**) ^11^B NMR spectrum of BSZ12 monomers.

BSZ12 and CPBCS were synthesized according to Figure 1. The structure of hyper-branched CPBCSs could be adjusted through changing the molar ratio of Si-H/C=C. Figure 2a shows the FTIR spectra of TMDS and BSZ12. The new peaks that appeared at 1092 cm^−1^ (C-B stretching) and 2875 cm^−1^ (-CH_2_- stretching) demonstrated that hydroboration occurred. However, the peak at 1338 cm^−1^ also indicated that the dehydrogenation reaction occurred between N-H and B-H, forming a B-N bond in BDMS. Hydroboration and dehydrogenation occurred simultaneously with the presence of N-H [38], and the resulting product (BSZ12) was a mixture of products from the two reactions (Figure 2b). This could be further verified by the ^1^H NMR spectrum (Figure 2c). The peaks range of 0.12–0.24 ppm (I) in ^1^H NMR spectrum is assigned to Si-CH_3_. The peaks at 0.50 (II) and 0.93 ppm (III) are assigned to Si–CH_2_-CH_2_– and methine in Si-CH(CH_3_)- groups. The peak at 1.24 ppm (IV) is assigned to -CH_3_ in B-CH(CH_3_)-). The peaks at 3.71–4.12 ppm (V) are assigned to the protons of the ethylene bond in B–CH_2_-, and the peaks at 5.88–6.19 ppm (VI) are assigned to -CH=CH_2_. Generally, the resulting polymer product was a mixture of α-adduct and β-adduct, and according to ^1^H NMR spectroscopy, the ratio of the α-adduct over the β-adduct was 5:3, irrespective of the monomer type.

^11^B NMR (Figure 2d) confirmed the formation of B-N bonds by peak resonances with chemical shifts in −7–3 ppm range (M), confirming the dehydrogenation between N-H and B-H. The peaks at 50 ppm (D^1^) and 61 ppm (D^2^) are assigned to B-CH(CH_3_)- and B-CH_2_-CH_2_-. It could be concluded that the BSZ12 monomer was a mixture of β-adduct, α-adduct, and products from dehydrogenation.

### 3.2. Synthesis of Crosslinked CPBCSs

Karstedt Catalyst Solution was used to catalyze the crosslinking reaction between BSZ12 and PNSZ due to its high catalytic activity, wide applicability, lack of by-products, and contribution to the thermal stability/flame retardancy of polymer materials [39,40]. The molar ratios of Si-H:C=C were 10:1, 5:1, and 3:1, and the samples were marked as CPBCS10-1, CPBCS5-1, and CPBCS3-1, respectively [15,41]. The Pt(0) concentration was fixed at 412 ppm to prevent catalyst poisoning by nitrogen atoms. Elemental analysis (Table 2) of the crosslinked products showed that the C and B content in the sample increased with increasing BSZ12. The boron content could be easily tuned by changing the ratios of PNSZ and BSZ12, and 1.37 wt% B content was achieved when Si-H:C=C was 3-1. All the samples showed a high oxygen content around 20 wt%, and the oxygen was not supposed to be present in the samples, since hydrosilylation reaction does not involve oxygen [42]. When we looked into the hydrosilylation catalyzed by Pt(0), we found that oxygen acts as a promoter and is involved in the formation of the intermediates [43]. The oxygen might be trapped in the materials, and at high temperatures, it could lead to the oxidation of Si-H and exist as Si-O-Si in the final resin system. The oxygen content could not be quantitatively controlled, and further structure changes at 300 °C lead to variability in nitrogen and oxygen content.

**Figure 3:**
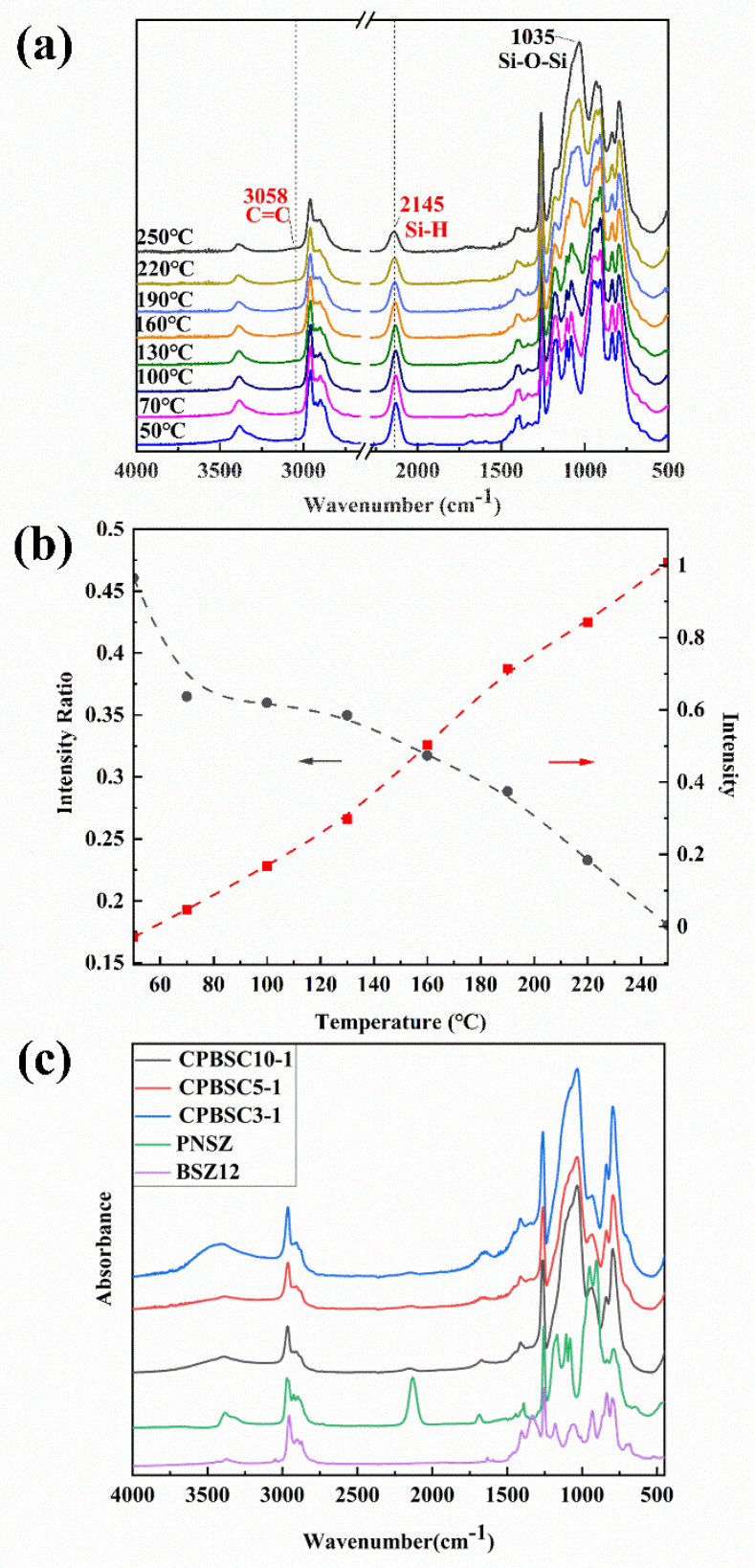
(**a**) FTIR spectra of CPBCS cured at different temperature for 2 h; (**b**) Peak intensity ratio of 2138 over 1260 cm^−1^ (gray), and 1035 over 1260 cm^−1^ (red) as a function of curing temperature; (**c**) FTIR spectra of CPBCS solids, BSZ12 and PNSZ were used to monitor the reaction.

The curing process of CPBCS was monitored by FTIR, as shown in Figure 3a. The intensity of the Si-H peak at 2145 cm^−1^ and the C=C peak at 3058 cm^−1^ decreased gradually as the curing temperature increased. The progress of the crosslinking reaction by hydrosilylation and dehydrogenation was analyzed by observing the corresponding peak intensity change with increasing temperature. The sharp and single peak at 1260 cm^−1^ (Si-CH_3_) was used as an internal standard since it did not participate in the crosslinking process. The intensity ratio *R* was calculated according to the following equation:

*R* = *h*_*x*_/*h*(Si-CH_3_) (2)

where *h_x_* is the peak height of the changing bond and *h*(Si-CH_3_*)* is the height of the Si-CH_3_ peak (1260 cm^−1^). The height of the Si-H and Si-O-Si peaks are 2138 cm^−1^ and 1035 cm^−1^.

Si-H intensity decreased with temperature due to hydrosilylation consumption. (Figure 3b) The appearance of Si-O-Si (1035 cm^−1^) was due to the oxygen participation in the hydrosilation reaction, and the intensity increased with temperature. The formation of Si-O-Si also consumed part of the Si-H bonds. Figure 3c shows the FTIR spectra of fully crosslinked CPBCS samples after heat treatment at 300 °C for 2 h. The disappeared Si-H bond (2145 cm^−1^) indicated that dehydrogenation between Si-H and Si-H or Si-H and N-H occurred, leading to higher crosslinking density.

### 3.3. Optical Properties and Surface Characteristics of CPBCSs

**Figure 4:**
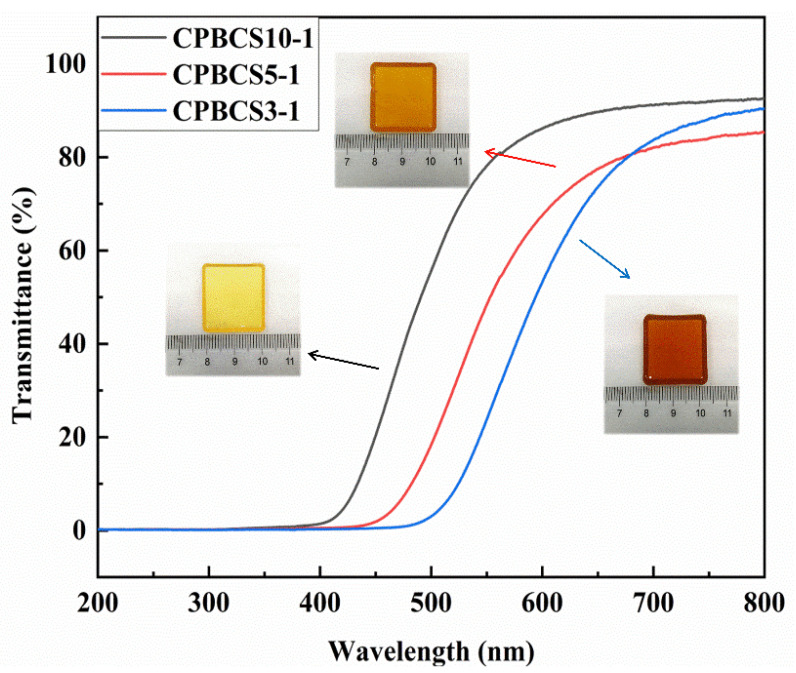
Digital images and UV-Vis transmission spectra of cross-linked CPBCS10-1, CPBCS5-1, and CPBCS3-1 polymers solids.

Figure 4 shows UV-Vis spectra and digital images of crosslinked CPBCS samples. The CPBCSs turned from light yellow to orange with increasing boron content, and the transmittance showed a bathochromic shift with increasing boron content. All the samples showed ≈0% transmittance below 360 nm because of the absorption of Pt from Karstedt Catalyst [44].

**Figure 5:**
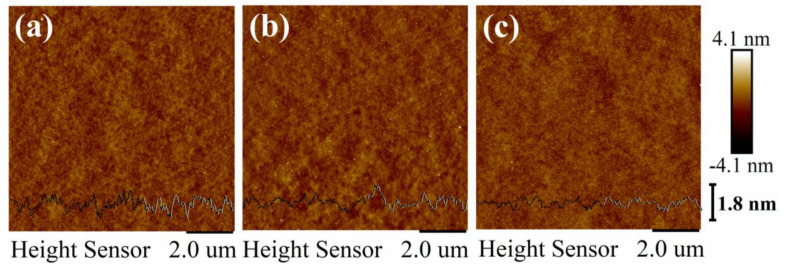
AFM height images and section analyses of the free (air) surface of crosslinking network (**a**) CPBCS3-1, (**b**) CPBCS5-1, and (**c**) CPBCS10-1 solids (upper data).

Figure 5 shows the typical topological images of the crosslinked CPBCSs. The morphologies revealed that CPBCSs have a smooth, crack-free surface (10 × 10 μm^2^). The mean square roughness (Rq) values of CPBCS3-1, CPBCS5-1, and CPBCS10-1 were 0.324 nm, 0.288 nm, and 0.325 nm, respectively. The results provided evidence that the organic and inorganic constituents of the hybrid polymer are distributed uniformly in the solids without any phase separation.

**Figure 6:**
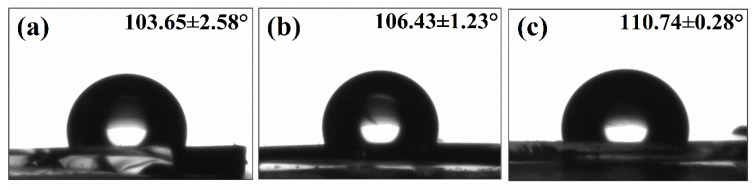
Static contact angles of water of CPBCS3-1, CPBCS5-1, and CPBCS10-1 polymers solids.

Static contact angles of water were measured, and the results are presented in Figure 6. The contact angles were determined to be 103.65° (CPBPS10-1), 106.43° (CPBCS5-1), and 110.74° (CPBCS3-1), which are comparable to that of extremely crosslinked silicone materials (109°) [45]. The hydrophobic surface is attributed to the formation of close-packed methyl groups at the surface resulting from the crosslinking of hybrid polymers with their chemical homogeneity as well as lower topographic roughness [45].

### 3.4. Thermal Stability of CPBCSs

**Figure 7:**
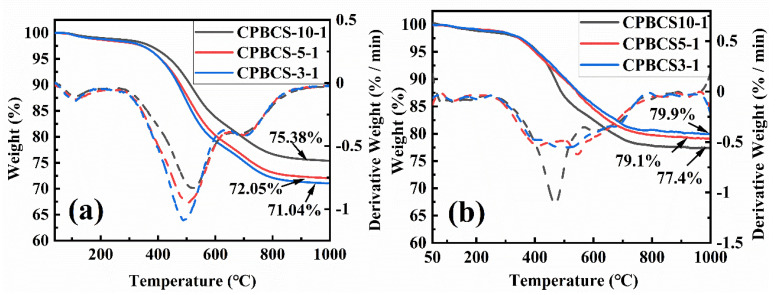
TG-dTG (derivative of TG curves) curves of CPBCSs: CPBCS3-1, CPBCS5-1, and CPBCS10-1 in argon (**a**) and in air (**b**).

Figure 7a shows the TG and 1st derivative of TG curves (dTG) in the argon atmosphere. Continuous mass loss was observed from 150 °C, and the mass loss before 400 °C was mainly attributed to the loss of low molecular weight dimers. The weight loss around 400–600 °C is mainly due to the cleavage of Si-CH_3_ bonds and Si-H bonds, and the weight loss in 600–800 °C is mainly due to the cleavage of the C-C framework in the form of methane and carbon dioxide gas [46]. The char yields at 1000 °C in argon were 75.38 wt%, 72.05 wt%, and 71.04 wt% for CPBCS10-1, CPBCS5-1, and CPBCS3-1, respectively. The char yields at 1000 °C in air were higher than those in argon, which is due to weight gain from oxidation. The high char yields indicated that CPBSC polymers could be used as preceramic polymers of SiBCN(O) [47].

Figure 7b shows the char yields at 1000 °C in air were 79.9 wt%, 79.1 wt%, and 77.4 wt% for CPBCS3-1, CPBCS5-1, and CPBCS10-1, respectively. BC units went through a similar oxidation behavior as BN(C), producing B_2_O_3_ phase. The B_2_O_3_ turned into liquid at high temperatures, which in turn prevents the oxidation of the inner materials by forming a protective layer [48]. B_2_O_3_ may react with glassy SiO_2_ to form stable borosilicate or SiO_2_-B_2_O_3_ binary melt, and the borosilicate glass can withstand up to 1600 °C. The dense borosilicate glass layer inhibits further oxidation [49,50]. Therefore, the char yield increased with boron content.

### 3.5. Flammability of CPBCSs

**Figure 8:**
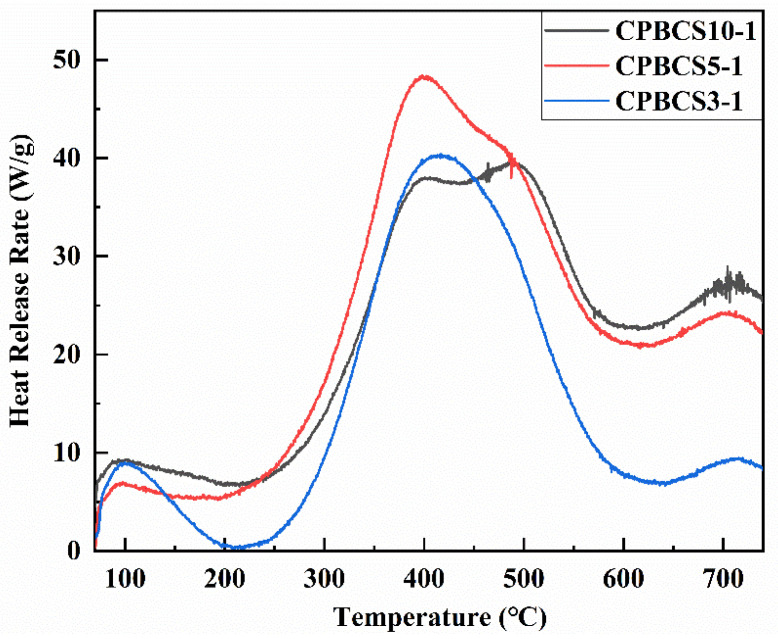
Heat release rate versus temperature in pyrolysis combustion flow calorimetry (PCFC) tests.

The flammability can be determined by the rate at which heat is released during burning. HRR vs. pyrolysis temperature plots of CPBCSs are shown in Figure 8, and the results are summarized in Table 3. The HRR profiles of CPBCSs shows multiple peaks. The first (the smallest) peak at 100–150 °C is related to the initial decomposition of the small molecules. The main peaks at 300–570 °C were due to the decomposition of Si-C and C-C, which is in agreement with the STA conducted in argon (Figure 7a).

The THR of CPBCSs is below 10 kJ/g, which is similar to that of flame-retardant polyimide (PI, sumHRC 38 J/g K, THR 6.7 J/g K) [51]. Compared with flame-retardant polyurethanes containing boron additives, CPBCSs have lower pHRR and sumHRC combustion peaks and higher peak thermal decomposition temperature [52].

The thermally stable B–N bond in the structure makes CPBCSs more stable. During the decomposition process, thermally stable bonds such as B–O–C are formed and further carbonized to form inorganic B_2_O_3_ or carbon, thus increasing the char yield and decreasing the heat release [50].

### 3.6. Mechanical Properties of CPBCSs

**Figure 9:**
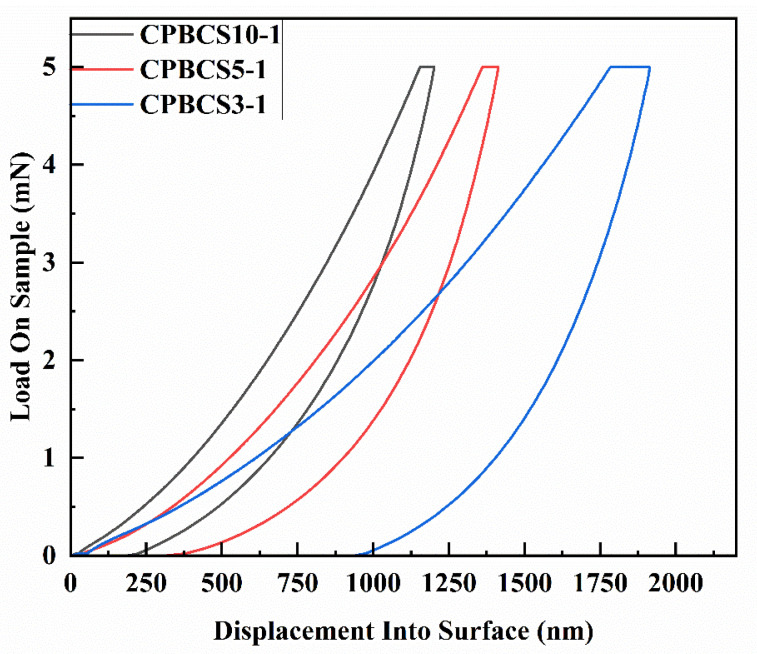
Nanoindentation curves of crosslinked CPBCSs.

The mechanical properties were quantitatively assessed using nanoindentation. Figure 9 shows the load–displacement curve of CPBCSs. When the load reached 5 mN, the displacements of CPBCS10-1, CPBCS5-1, and CPBCS3-1 were 1155 nm, 1362 nm, and 1785 nm, respectively. After holding for 5 s under a constant force of 5 mN, the displacement of the three samples increased to 1201 nm, 1412 nm, and 1913 nm, respectively. The low indentation depth increases during the holding period indicated that all the CPBCS are hard and resistant to shape change. The elasticity decreased with increasing boron content, which is mainly attributed to the presence of free space between PNSZ chains for deformation, absorbing more energy. The reduced modulus and hardness of the crosslinked polymers are presented in Table 4. CPBCS3-1 showed the lowest reduced modulus and hardness, while CPBCS10-1 showed the highest reduced modulus and hardness.

### 3.7. An Insight into CPBCSs’ Structure

When BSZ12 and PNSZ undergo hydrosilylation reaction, the Si-H from PNSZ could not be completely consumed, since excessive Si-H groups were employed in the synthesis process. The use of BSZ12 also introduced space between crosslinking points. Further heat treatment at 300 °C for 2 h led to degeneration reactions between Si-H and Si-H, and Si-H and N-H. However, in CPBCS 3-1, the crosslinking density is high, and the Si-H and N-H groups on the chains cannot react easily due to steric hindrance. A similar situation exists in CPBCS5-1. However, when it comes to CPBCS10-1, the PNSZ chains between crosslinking points are long enough to entangle with each other and contribute to dehydrogenation reaction at 300 °C. The additional dehydrogenation reaction in CPBCS10-1 contributes to a higher crosslinking density than that of CPBCS5-1 and CPBCS3-1, showing better mechanical properties. At the same time, with the increase of BSZ12, the boron and B-C structure content in CPBCS also increase, which contributes to the high-temperature oxidation stability of the material and provides more reliable flame retardant and fire resistance.

## 4. Conclusions

Novel polyborosilazanes were successfully fabricated through a two-component route via hydrodilylation. The boron content and polymeric structure could be readily tuned through changing the Si-H/C=C ratios. The solventless preparation process relieves the pressure on the environment and reduces the waste of resources. The increase of boron content and B-C structure in CPBCSs can form SiO_2_-B_2_O_3_ binary melt and borosilicate glass, forming a dense oxide layer to reduce its heat release, which hinders heat transfer to the underlying polymer. CPBCSs showed good thermal stability and thermal oxidation performance, and they also showed good flame retardancy with an HRR lower than 50 W/g. The as-prepared polyboronsilazanes could be potentially used as preceramic polymers to produce SiBCN(O) ceramic components for high-temperature applications and flame retardant coatings to protect materials against fire or oxidation.

## Figures and Tables

**Table 1:** Ratios of multifunctional boron hybrid silazane monomers (BSZ12) and poly[imino(methylsilylene)] (PNSZ) for synthesizing cross-linked boron-containing silicone polymers (CPBCSs).

Sample	BSZ12 (g)	PNSZ (g)	Molar Ratio (Si-H/C=C)
CPBCS10-1	1.2962	4.000	10-1
CPBCS5-1	2.5924	4.000	5-1
CPBCS3-1	3.2192	3.000	3-1

**Table 2:** Polymer element content of crosslinked polymer CPBCS solids.

Samples	CPBCS10-1	CPBCS5-1	CPBCS3-1
Si	44.30%	35.40%	33.05%
C	24.50%	30.10%	32.90%
O	20.22%	19.80%	20.23%
H	7.55%	8.35%	8.65%
N	2.80%	5.33%	3.80%
B	0.63%	1.02%	1.37%

**Table 3:** Data obtained from PCFC tests.

Sample	pHRR_1_ (W/g)	Tp_1_ (°C)	pHRR_2_ (W/g)	Tp_2_ (°C)	pHRR_3_ (W/g)	Tp_3_ (°C)	sumHRC (J/g K)	THR (kJ/g)
CPBCS3-1	8.9	45.5	40.3	356.5	-	-	40	7.5
CPBCS5-1	6.9	46.0	48.3	342.1	41.1	436.0	43	9
CPBCS10-1	9.1	45.5	37.9	344.0	39.5	457.5	43	6.2

**Table 4:** Hardness and modulus of CPBCSs.

	CPBCS10-1	CPBCS5-1	CPBCS3-1
**Reduced Modulus (GPa)**	2.32 ± 0.08	1.93 ± 0.09	1.40 ± 0.10
**Hardness (GPa)**	0.30 ± 0.02	0.18 ± 0.01	0.09 ± 0.01

## Data Availability

The data presented in this study are available on request from the corresponding author.
